# Prospects for Bioinspired Single-Photon Detection Using Nanotube-Chromophore Hybrids

**DOI:** 10.1038/s41598-019-39195-1

**Published:** 2019-03-01

**Authors:** François Léonard, Michael E. Foster, Catalin D. Spataru

**Affiliations:** Sandia National Laboratories, Livermore, CA 94551 USA

## Abstract

The human eye is an exquisite photodetection system with the ability to detect single photons. The process of vision is initiated by single-photon absorption in the molecule retinal, triggering a cascade of complex chemical processes that eventually lead to the generation of an electrical impulse. Here, we analyze the single-photon detection prospects for an architecture inspired by the human eye: field-effect transistors employing carbon nanotubes functionalized with chromophores. We employ non-equilibrium quantum transport simulations of realistic devices to reveal device response upon absorption of a single photon. We establish the parameters that determine the strength of the response such as the magnitude and orientation of molecular dipole(s), as well as the arrangements of chromophores on carbon nanotubes. Moreover, we show that functionalization of a single nanotube with multiple chromophores allows for number resolution, whereby the number of photons in an incoming light packet can be determined. Finally, we assess the performance prospects by calculating the dark count rate, and we identify the most promising architectures and regimes of operation.

## Introduction

The human eye is a fantastic photodetector: it works at room temperature in a complex biological environment, and yet it is able to detect single photons^1^. On the other hand, much work has been devoted to the fabrication of synthetic single-photon systems^2^ such as superconductor nanowire detectors^3–5^ and avalanche photodiodes^6,7^. Each of these approaches provides advantages for single-photon detection, but also suffer from limitations. Thus, new photodetector modalities that could circumvent or alleviate some of these limitations are of interest. One such approach is to use nanomaterials and concepts from solid-state devices to realize bioinspired detectors.

Previously, a new type of photodetector based on carbon nanotubes functionalized with chromophores^8–10^ demonstrated photoresponse from the UV to the Visible using synthetically tailored photoactive chromophores^11^. These experiments, done using relatively high-intensity light sources, suggested sensitive photoresponse based on the small nanotube cross-section, but to date, single-photon detection with this system has not been demonstrated. Thus, an open question remains: can this system be used for single-photon detection? Hints that this is possible come from experiments in single molecule detection where large changes in CNT conductance were measured upon absorption of individual molecules^12,13^.

In this manuscript, we present detailed quantum transport simulations of realistic field-effect transistors using chromophore-functionalized carbon nanotubes as the active channel material. We study the case when a single photon (or a discrete number of photons) is absorbed by the chromophores/molecules. We show how this leads to modulation of the current in the carbon nanotube (CNT), and establish the parameters that determine the strength of the response. We consider the case of multiple molecules on a CNT and demonstrate that photon number resolution is possible with careful engineering of the molecular arrangement. We use *ab initio* calculations coupled with tight-binding quantum transport simulations to study the specific case of the azobenzene molecule Disperse Red 1 (DR1). This shows the development and integration of a hierarchy of models (i.e. *ab initio* to semi-classical) that can handle a broad range of device dimensions, molecular densities, molecular arrangements, and modes of operation. Finally, we evaluate the dark count rate and establish the most favorable operation regimes in terms of the integration time, strength of the response, and the number of CNTs in the transistor.

The process of human vision begins with the absorption of a single photon by the molecule retinal, which then undergoes a conformation change from the *cis* to the *trans* state. This change in shape causes retinal to be expulsed from its host protein, triggering a long chain of events that involves different enzymatic reactions and recycling of products^14^. It is a remarkable process that is not only sensitive to single photons but is also repeatable over many years. Considering these amazing properties, one may wonder if mimicking and simplifying the process can lead to the synthetic realization of photodetectors with enhanced performance.

Another important aspect of the human vision system is the presence of rods and cones which provide low-light gray vision and daytime color vision, respectively. The color sensitivity of cones comes from the different conformational orientations of retinal in the host protein; unfortunately, cones lack the sensitivity of rods because each cone is connected to a neuron, while multiple rods connect to the same neuron thus increasing the probability of a neuron firing. A design improvement would be to combine the single-photon sensitivity of rods with the color discrimination of cones.

**Figure 1:**
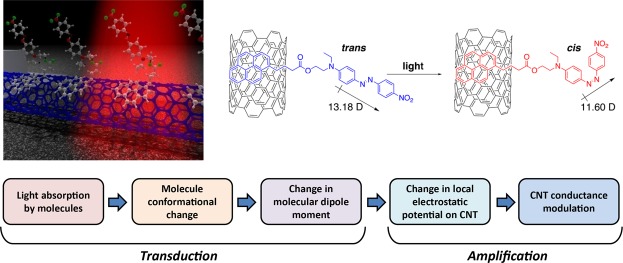
Illustration of the concept and the processes. (**a**) Chromophores are attached to the surface of a CNT using a non-covalent linker. Here the chromophore is Disperse Red 1 and the linker pyrenebutyric acid. (**b**) Upon light absorption, the chromophore undergoes a conformational change that changes the dipole moment. Here the case of DR1-PB is illustrated. (**c**) The processes can be divided into transduction where the light is converted into a molecular conformational change, and amplification, where the molecular conformational change leads to modulation of the CNT conductance.

One approach to simplifying the process while imparting color discrimination and sensitivity is shown in Fig. 1. There, a semiconducting single-wall carbon nanotube is functionalized with chromophores. Upon light absorption, the chromophore undergoes a conformational change accompanied by a change in its dipole moment and direction. This in turn changes the electrostatic potential on the CNT, which impacts its electronic transport properties. When operated as a field-effect transistor, the conductance in the dark can be adjusted by several orders of magnitude, providing a way to probe the best regime of operation. In this manuscript, we focus on the electronic transport part of the process, i.e. we assume that the photon has been absorbed and the molecule has changed conformation. We consider CNTs because the quasi-one-dimensional transport can be entirely affected by the localized potential perturbation from the chromophore. Two-dimensional materials could also be used, but their lateral size would have to be made small enough to make the chromophore perturbation of significance on the total electronic transport.

The efficiency of the transduction process (i.e. light absorption) is also of much interest and depends on the particular geometry. For example, for a plane wave photon field of flux Φ, the number of photons absorbed by a single molecule in time τ is given by Φτσ where *σ* is the molecule optical absorption cross-section. Using typical values of *σ* ~ 1 Å^2^ and considering a 1 nW light source with 1 eV photons focused to a spot of size 1 μm^2^, we obtain about 100 photons absorbed per second. This rate makes the system amenable to fundamental science experiments. On the other hand, because of the small *σ* the efficiency is low (about 10^-6^% for the case above) so for efficient detection of infrequent single photons other architectures are required. One possibility is to put the device inside of a waveguide, which has recently been theoretically shown to lead to high detection efficiency in model single molecule systems^15^. Another possibility is to make dense three-dimensional arrays of nanometric devices, guaranteeing that the photon will be absorbed by one of the devices in the array.

We also note that the CNT also absorbs strongly over a broad range of energies, but for weak molecule-CNT coupling the optical absorption of the molecule itself is not affected by the presence of the CNT^16^, so the molecular absorption cross-section remains the same. Furthermore, it is possible to choose molecules with absorption spectra that do not overlap with the main CNT excitonic absorption lines. Also, while there could be direct photocurrent generation due to absorption by the CNT, this process is inefficient due to the need to dissociate the exciton and collect the carriers, and usually leads to small photocurrents in CNT transistors like the ones considered here^17^; this is in agreement with control experiments comparing the photoresponse of CNT transistors with and without molecules^8,11^. Finally, other mechanisms, such as photoinduced charge transfer, could also lead to photodetection, and we anticipate that the formalism presented here would be useful to analyze such cases as well.

## Methods

### Tight-binding NEGF

**Figure 2:**
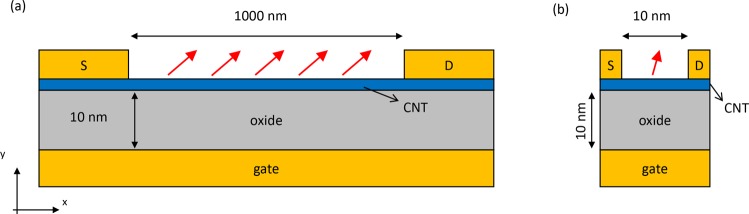
Sketch of the simulated CNT transistors functionalized with dipolar molecules. The red arrows represent the change in molecular dipole moment upon light absorption. (**a**) Long channel transistor. (**b**) Short channel transistor.

Our computational approach for CNT electronic devices has been described in detail in previous publications^18,19^. As illustrated in Fig. 2 the simulated field-effect transistor consists of a (17, 0) semiconducting CNT (radius = 0.66 nm) connected to source and drain electrodes. The CNT sits on a SiO_2_ dielectric of 10 nm thickness, below which the gate electrode is located. The CNT is modeled using a tight-binding model with a nearest-neighbor interaction of 2.5 eV. In this model, the CNT bandgap is 0.5 eV. The CNT is separated from the dielectric and the metal contacts by a van der Waals distance of 0.3 nm. We take the CNT workfunction to be 4.5 eV and the metal workfunction 5.2 eV, which is representative of Pd contacts, the common metal used to make low-resistance contacts to CNTs.

We first obtain the band-bending along the CNT using a self-consistent calculation between the charge on the CNT and the electrostatic potential in the whole device. The charge on the CNT is calculated using the non-equilibrium Green’s function (NEGF) approach, while the potential is obtained by solving Poisson’s equation using finite difference. Once the potential along the CNT is obtained, we use NEGF to calculate the device conductance.

This tight-binding NEGF approach allows us to simulate long channel devices with thousands of atoms, as illustrated in Fig. 2a. This figure shows a schematic representation of a one micron channel CNT device, functionalized with many molecules. Upon absorption of a single photon, with some probability, a molecule will undergo a conformational change, and hence a change in dipole moment and/or direction. We model the molecular dipoles in the states before and after the conformational change as perfect dipoles, with the change in dipole moment given by ${\rm{\Delta }}\overrightarrow{\mu }$. Due to this change in dipole moment, the change in the electrostatic potential on the CNT is given by

1 $${\rm{\Delta }}V(\overrightarrow{r})=\frac{1}{4\pi {\varepsilon }_{0}}\frac{{\rm{\Delta }}\overrightarrow{\mu }.(\overrightarrow{r}-\overrightarrow{r}^{\prime} )}{|\overrightarrow{r}-\overrightarrow{r}^{\prime} {|}^{3}}$$

where $\overrightarrow{r}$ is a vector describing the positions of the carbon atoms on the CNT and $\overrightarrow{r}^{\prime}$ is the dipole position. This change in electrostatic potential is added as a diagonal contribution to the CNT Hamiltonian, and the NEGF calculations compare the conductance with and without the potential perturbation.

### Tight-binding NEGF with ab initio input

To verify the validity of the above model Eq. (1) for the molecular dipoles, we performed ab initio calculations of the molecule DR1-PB and compared the potential obtained from ab initio with Eq. (1). DR1-PB was chosen based on its relevance to the previous experimental literature^11^. The dipole moments and vectors were obtained from density functional theory (DFT) calculations of the isolated molecule (*cis* and *trans*); the molecular orientations of DR1-PB were extracted from the optimized periodic systems (*trans*-DR1-PB@CNT and *cis*-DR1-PB@CNT; ten repeat units of a (17, 0) CNT were used (680 atoms)). This allowed for the determination of the dipole moments and vectors of DR1, in the *cis* and *trans* states, with respect to the orientation of the CNT. The calculations on the isolated molecule were performed at the PBE/6-31G(d, p) level of theory using Gaussian 09^20^; the periodic systems were optimized (atom positions and cell dimension parallel to the CNT) using the self-consistent-charge density functional tight binding (SCC-DFTB) method including third-order expansion of the DFT total energy (DFTB3)^21^ and Grimme’s empirical van der Waals dispersion^22^ corrections. This approach allowed for reasonable molecular orientations to be achieved with modest computational expense; the calculations were performed using the DFTB+ software package^23^. In addition, single-point periodic PBE calculations were performed on the optimized geometry to obtain the electrostatic difference potentials, which were used as input for our tight-binding NEGF model (see discussion below). These calculations were performed using Atomistix ToolKit version 2016.3, QuantumWise A/S^24,25^.

## Results

### Basic considerations

**Figure 3:**
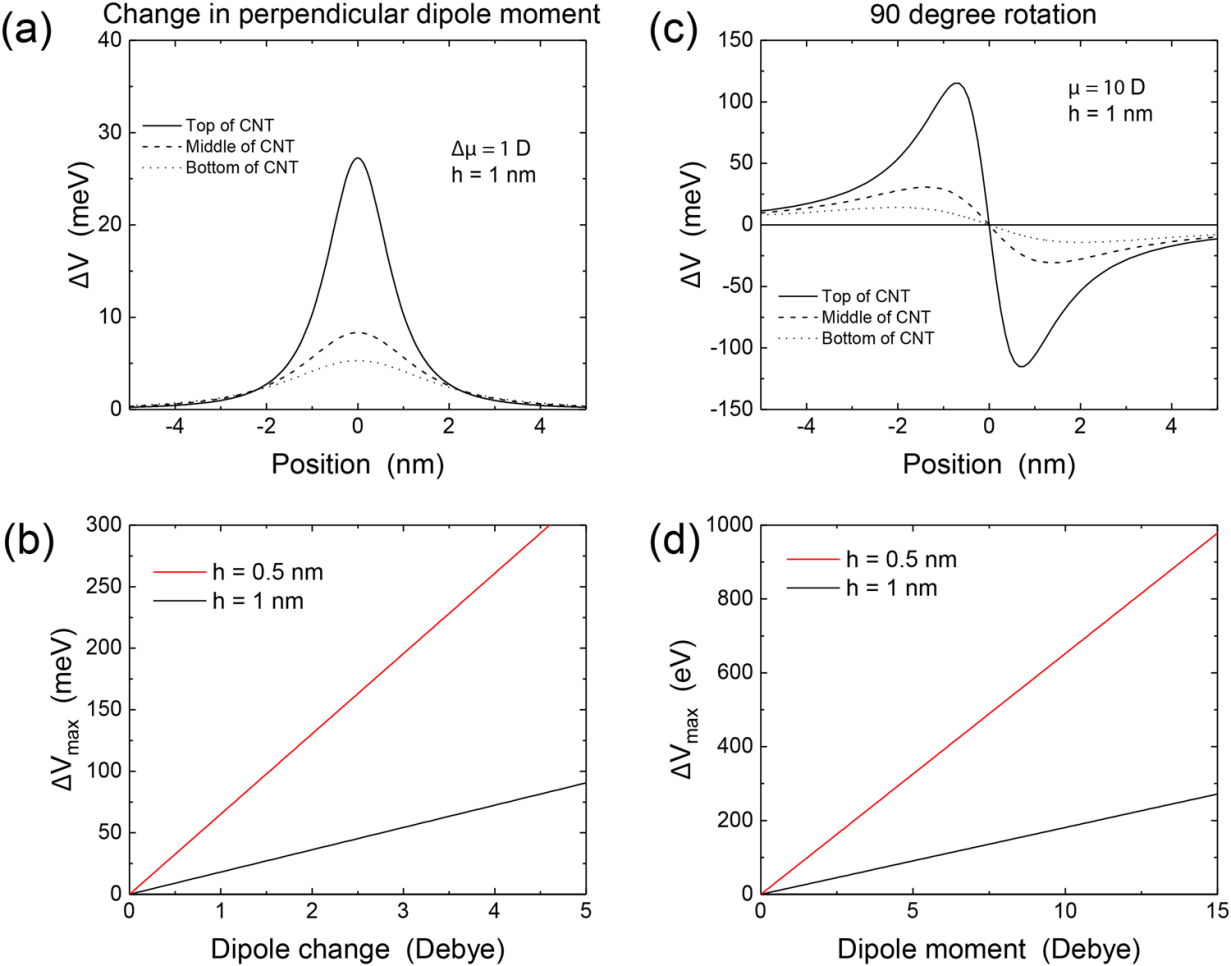
Change in electrostatic potential on the CNT due to changes in the molecular dipole moment. Panels (a) and (b) are for the case where the dipole stays oriented in the direction perpendicular to the CNT axis and undergoes a change in magnitude upon light absorption. Panels (c) and (d) are for the case where the dipole magnitude stays fixed and the dipole rotates from a perpendicular orientation to an orientation along the CNT axis.

We first begin by discussing the basic general properties of the dipole system and its potential impact on the CNT electronic transport. We consider a molecule of dipole moments ${\overrightarrow{\mu }}_{1}$ in the *trans* (ground) state and ${\overrightarrow{\mu }}_{2}$ in the *cis* (meta-stable) state, with the change in dipole moment given by ${\rm{\Delta }}\overrightarrow{\mu }={\overrightarrow{\mu }}_{2}-{\overrightarrow{\mu }}_{1}$. For example, our *ab initio* calculations discussed below show that for DR1, ${\overrightarrow{\mu }}_{1}=11.6D$ and ${\overrightarrow{\mu }}_{2}=6.1D$ (see refs^11,26^ as well). This indicates that the magnitude of the dipole moments is about 10 D, while the change in magnitude between the two states is several Debye. Figure 3a show the electrostatic potential along the CNT for a dipole change of 1 Debye oriented perpendicular to the CNT, and with the dipole located a distance *h* = 1 nm from the surface of the CNT. The potential perturbation extends over ~2 nm and varies in amplitude between 125 meV at the CNT surface closest to the molecule and 45 meV at the bottom surface. For simplicity, we use the potential in the middle of the CNT as the measure of the potential perturbation, and plot in Fig. 3b the amplitude of the perturbation as a function of the dipole moment. The figure demonstrates that the perturbation can approach or even exceed the room-temperature thermal energy (25 meV) when reasonable changes in dipole moments are considered. The effect can be further enhanced by using molecules that are closer to the CNT; indeed, a five-fold increase in the amplitude of the perturbation is obtained with a separation of 0.5 nm compared to 1 nm.

Much larger changes in the potential can be obtained by rotating the dipole instead of changing the magnitude of the dipole moment. This is because the magnitude of the dipole moment is typically much larger than the magnitude of the change in dipole moment, as discussed above. For example, Fig. 3c shows that rotating a 10 Debye dipole moment by 90 degrees (from being oriented perpendicular to the CNT to being oriented along the CNT axis) leads to changes in the potential that can reach 100 meV for a 1 nm separation. This can be enhanced to several hundred of meVs by decreasing the spacing between the molecule and the CNT (Fig. 3d).

### Long channel device

**Figure 4:**
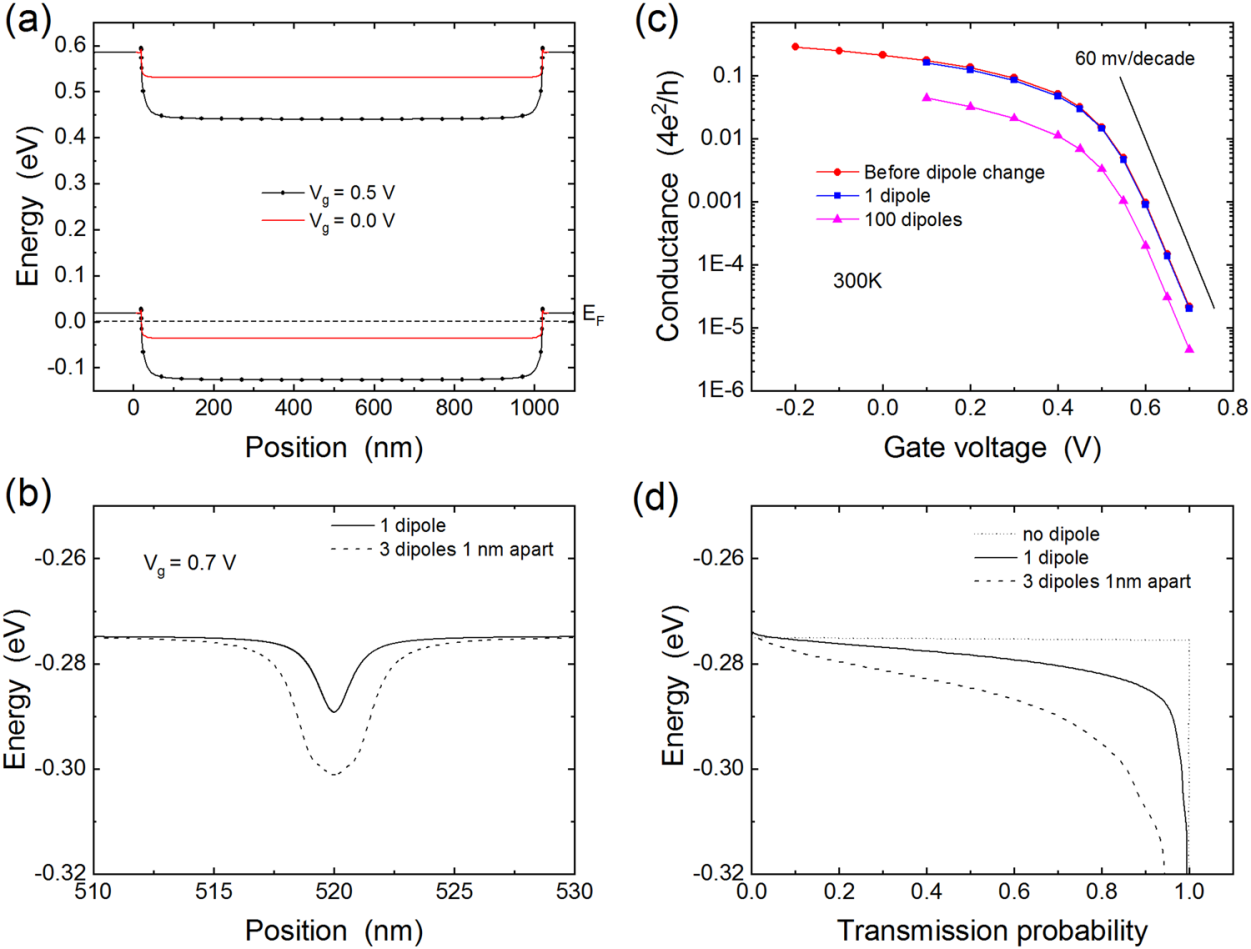
(**a**) Band-bending before the dipole change for two values of the gate voltage. (**b**) Perturbation to the valence band edge in the middle of the FET channel due to switching of one and three dipoles. The change in dipole moment is 1 Debye oriented perpendicular to the CNT. The molecules are 1 nm from the CNT. The gate voltage is 0.7 V. (**c**) Transfer characteristics of the CNT-FET before and after photoswitching of the chromophores. (**d**) Transmission probability for electrons as a function of energy. The vertical axis is aligned with panel (c) to show the origin of the transmission reduction.

**Figure 5:**
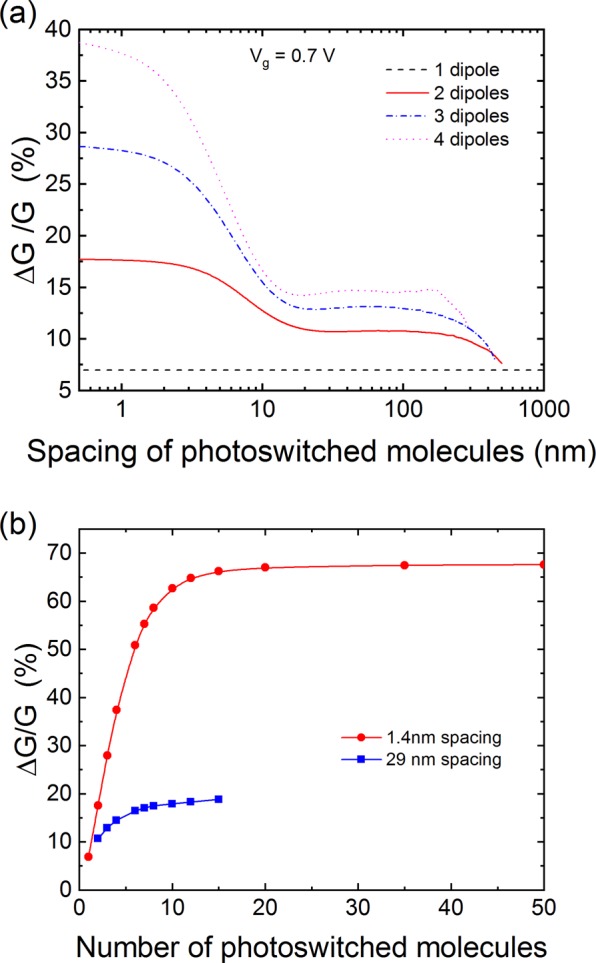
Number resolution with a long channel CNT-FET for a dipole change of 1 D. (**a**) Change in conductance as a function of the separation between photoswitched molecules. (**b**) Change in conductance as a function of the number of photoswitched molecules for the case where neighbor molecules are photoswitched (1.4 nm spacing) or when the molecules are initially positioned far apart (29 nm spacing).

Figure 4a shows the calculated band-bending and transfer characteristics of the long channel device obtained using the TB-NEGF approach. The behavior is typical of p-type FETs due to the p-type doping of the CNT under the contacts. This p-type doping is due to charge transfer from the metal to the CNT because of the large metal workfunction compared with that of the CNT. By applying a positive gate voltage, the bands bend down in the channel, increasing the barrier for hole injection; this decreases the conductance and turns off the FET. To illustrate the impact of photoswitching of chromophores, we added a dipole change of 1 Debye to a molecule located 1 nm from the top surface of the CNT. (As discussed above, other types of molecular transformations could lead to larger effects. Here we deliberately chose a conservative case; if such a case is promising for single photon detection, other effects would be even more promising). As shown in Fig. 4b this introduces a dip in the valence band profile at the location of the molecule, in line with the potential change of Fig. 3a. We calculated the room temperature transfer characteristics of the CNT-FET as a function of the gate voltage with and without the dipole change. In the dark, the device behaves as a p-type FET transistor with a subthreshold swing approaching the thermal limit of 60 mV/decade (Fig. 5c shows). Upon switching one chromophore, we obtain a reduction of the conductance of about 7%. When one hundred chromophores separated by 1 nm are simultaneously switched, a much stronger change in conductance is observed. The origin of the reduction in conductance at a given gate voltage originates from electron scattering on the potential perturbation caused by the dipole change. Indeed, Fig. 4d shows the transmission probability near the edge of the valence band, with the vertical axis of the graph aligned with that of Fig. 4b. In the absence of the dipole change the transmission probability is zero inside the bandgap and jumps to one at the band edge. In contrast, when the potential perturbation is present, a significant reduction of the transmission probability is seen near the band edge, extending a few tens of meV below the band edge.

### Number resolution

The large difference in the conductance reduction between one and one hundred photoswitched molecules presented in Fig. 4c suggests a concept for a photon number-resolving detector where the strength of the signal depends on the number of absorbed photons. To test this idea, we calculated the change in conductance as a function of the number of photoswitched molecules on the CNT. However, in the absence of antenna effects, there is no guarantee that neighbor molecules will be photoswitched; for example, if two photons are absorbed by two chromophores, the two chromophores could be any two of the many chromophores on the CNT. (We recently became aware of a calculation that identifies antenna effects that favor neighboring dipole excitation^27^). To take this possibility into account, we considered the situation where the photoswitched chromophores are uniformly separated by a distance *l*, and we calculated the change in conductance as a function of *l*. (Note that the distance between photoswitched chromophores is not the same as the distance between the chromophores. For example, the chromophores could be 1 nm apart on the CNT, but two photons could switch two chromophores that are 100 nm apart). While in general there are many configurations where the photoswitched chromophores are not evenly spaced, the neighbor and evenly spaced results already allow us to reach general conclusions. Figure 5a shows the calculated change in conductance as a function of the separation between the photoswitched dipoles for up to four photons. As indicated above, for a single photon the change in conductance is about 7%. This is independent of the location of the photoswitched chromophore if it is located in the region of the channel where the bands are flat. If the photoswitched chromophore is located too close to the contacts where the bands bend upwards it does not add a perturbation below the lowest point of the valence band edge and therefore does not change the conductance. This implies that either the CNT should not be functionalized near the contacts, or light should be directed away from the contacts. Figure 5a shows that if the photons are absorbed by neighbor molecules (i.e. small separation of the photoswitched molecules) a clear demarcation exists between 1, 2, 3, and 4 photons. However, when the distance between photoswitched molecules is increased, the change in conductance decreases for all cases with more than one photon. In particular, the change in conductance for the 2, 3, and 4 photon cases start to overlap; for example, if three photons are absorbed by three molecules 20 nm apart, the change in conductance is the same as two photons absorbed 9 nm apart. Note however that a plateau for each photon number is found when the separation is larger than 20 nm. Thus, there are two regimes where number resolution might be possible. The first regime requires that neighbor molecules are always predominantly photoswitched over separated molecules. Such a situation may be favored by engineering the molecule interactions such that antenna effects favor neighbor excitation^27^. The second regime is when the separation of molecules on the CNT is chosen to be larger than 20 nm. In this case, the response will always be given by the plateau region of Fig. 5a. To further illustrate the behavior in these two regimes, we show in Fig. 5b the change in conductance as a function of the number of absorbed photons. In both cases, a few photons can be resolved (<10) until the change in potential saturates as the number of photoswitched molecules increases. Note that in the case of the large initial spacing of chromophores, only a small number of molecules can be positioned along a given channel length, which sets an upper limit on the number of photons that can be resolved with a single CNT.

**Figure 6:**
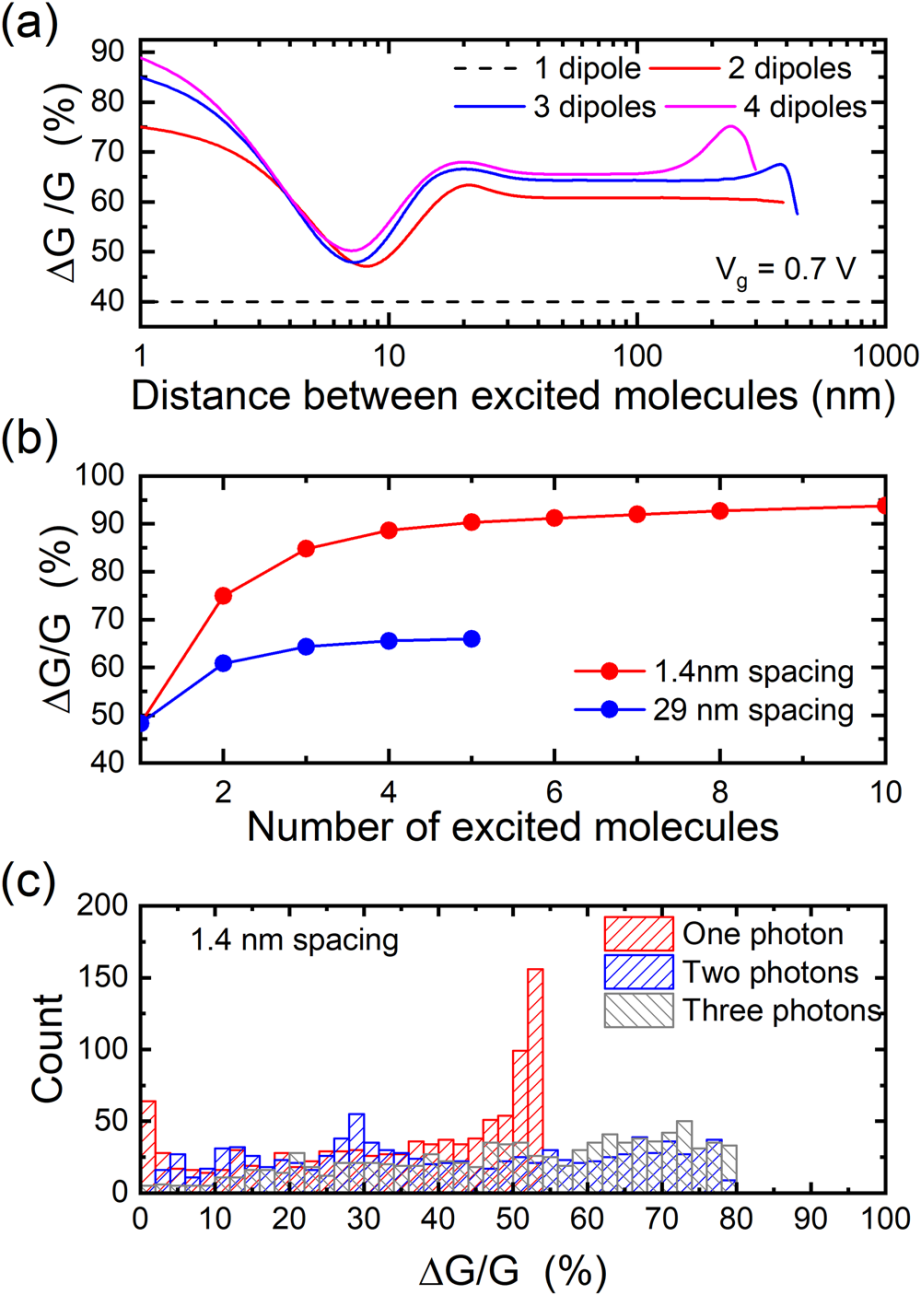
Number resolution with a long channel CNT-FET for a dipole change of 10 D. (**a**) Change in conductance as a function of the separation between photoswitched molecules. (**b**) Change in conductance as a function of the number of photoswitched molecules for the case where neighbor molecules are photoswitched (1.4 nm spacing) or when the molecules are initially positioned far apart (29 nm spacing). (**c**) Distribution of conductance changes for random rotations of the molecular dipole moments.

We also considered number resolution in the case of the 90 degree rotation of the molecular dipole upon light absorption. In this case, the impact of a single dipole is much larger (c.f. Fig. 3), leading to a 40% decrease of the conductance. While this increases the likelihood of detecting a single photon, it reduces the number of photons that can be resolved since only 60% of the total conductance is available to fit other states (compared with 93% for the perpendicular dipole case). This effect can be seen in Fig. 6 where we plot the change in conductance as a function of the photoswitched dipole separation. Just like in the case of the change in the perpendicular dipole moment, number resolution is possible if neighbor molecules are photoswitched, but it is only for a separation of at least 30 nm between molecules that a more likely scenario emerges. However, the number resolution is limited, as shown in Fig. 6b, because of the already large change in conductance for a single dipole change. Indeed, less than 5 photons can be resolved. The results for the perpendicular and parallel cases show that a tradeoff exists between the sensitivity of the detector and the number resolution.

Another challenge is the potential random positioning of the molecules on the CNT, which could lead to random changes in the dipole moments. For molecules such as azobenzenes, the angle between the initial dipole orientation and the final dipole orientation is always the same, but the angle between the initial molecular dipole moment and the CNT axis could vary depending on the positioning of the molecules. This is not a concern in the case of a purely perpendicular change in dipole moment since the dipole moment will always be perpendicular to the CNT axis no matter the location of the molecule. On the other hand, a more general situation would not be so ideal. For example, the 90-degree rotation is an extreme case, since the rotation from a perpendicular dipole to a longitudinal dipole could occur in different directions depending on the molecule position. To assess the importance of this effect on photon number resolution, we performed 1000 simulations of random rotation directions for one, two, and three photons (3000 simulations total). The distributions of conductance changes for each case are shown in Fig. 6c and indicate that each of the three cases has a broad spread of conductance changes. In addition, there is a significant overlap between the conductance distributions, making number resolution more difficult. This result emphasizes the need for controlled molecular organization on CNTs. The simplest approach is to ensure that all moleculles have a change in the perpendicular component of their dipole moment. Recent experimental work has demonstrated this to be the case for collections of azobenzene molecules on CNTs^28^. Another approach could be to graft the chromophores onto polymers that wrap around the CNT^29^.

### Tight-binding NEGF with ab initio input

**Figure 7:**
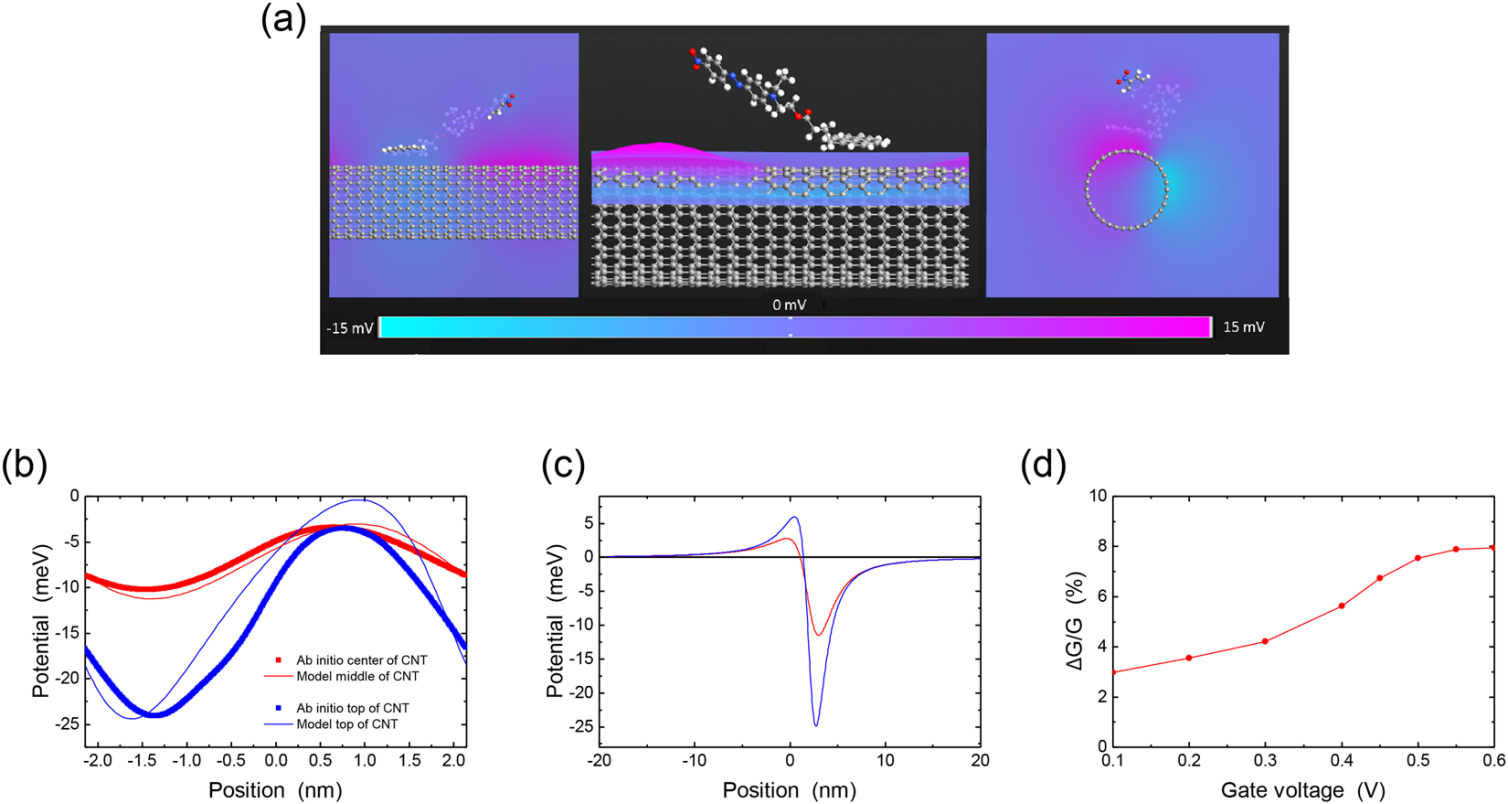
(**a**) *Ab initio* calculation of the interaction between Disperse Red 1 and a semiconducting CNT. The equilibrium structure of the *trans* configuration of DR1-PB on the CNT is shown. The unit cell is repeated in all three directions. The color map shows the electrostatic potential difference between the *trans* and *cis* configurations. (**b**) The electrostatic potential from the color maps of panel (a) extracted for two positions along the CNT. The *ab initio* results are compared to the model dipole potential of Eq. (1). (**c**) The potential of a single dipole extracted from the results of panel (a). (**d**) Change in conductance as a function of gate voltage using the single dipole potential of panel (b).

To test the above ideas on more concrete molecules, we used *ab initio* simulations to study the properties of the molecule Disperse Red 1 (DR1) attached to the surface of a (17, 0) CNT through a pyrenebutyric acid linker (PB). DR1 is a well-known azobenzene with a photo-induced transition between the *trans* ground state and the *cis* meta-stable state^30^. In addition, it is known to possess a large change in dipole moment upon isomerization; this was also confirmed from *ab initio* calculations in the case of DR1-PB attached to CNTs as previously discussed^11^. However, these previous calculations did not consider the impact of photoisomerization on the CNT electronic transport. To address this aspect, we compared the electrostatic potential on the CNT for the *trans* and *cis* configurations, and used the difference between the two as an input to the TB-NEGF calculations. Figure 7a shows the relaxed SCC-DFTB3-D3 structure of the *trans* configuration of DR1-PB on the CNT, and the electrostatic potential difference between the *trans* and *cis* configurations near the top of the CNT (i.e. closer to the molecule). We extracted this potential along lines at the top of the CNT and in the middle of the CNT as shown in Fig. 7b. At the top of the CNT, the potential difference between *trans* and *cis* has an amplitude of about 25 meV, which decreases to 10 meV in the center of the CNT. (We note that other changes in conformation are possible, such as rotation of the dipole; to fully explore these possibilities would require dynamic quantum simulations in the presence of light absorption, which is beyond the scope of this paper. Our calculations are meant to illustrate how ab initio calculations can be used to connect with the NEGF simulations).

Because the *ab initio* calculations use a periodic unit cell repeated in all three directions, we constructed an array of model dipoles using Eq. (1) for the potential of a single dipole. The magnitude and direction of the dipole change were obtained from the *ab initio* simulations, leaving only the distance between the dipoles and the CNT as the fitting parameter. Figure 7b shows a fit of the potential due to a model dipole array to the *ab initio* potential; good agreement was obtained for dipoles located 1.4 nm above the CNT. The model dipole parametrization from the *ab initio* simulations of the arrays allows us to obtain the potential for switching of a single dipole. This potential is shown in Fig. 7c and displays a maximum amplitude of 25 meV near the molecule with a width of a few nanometers. This extra potential causes scattering of the electrons in the CNT as discussed above and leads to a reduction of the conductance. Indeed, when input into the TB-NEGF calculations, the potential difference between *trans* and *cis* gives a change in conductance between 3% and 8% depending on the gate voltage, as illustrated in Fig. 7d.

Previous experiments on photoswitching of functionalized CNTs have measured large conductance changes^8,9,11^ for light intensities where up to thousands of molecules were photoswitched. While the specific mechanisms still must be determined for these different systems, the results of Fig. 5 indicate that even if the change in conductance for a single molecule is only a few percent, switching many molecules can lead to large conductance changes, as observed in the experiments.

### Short channel devices

**Figure 8:**
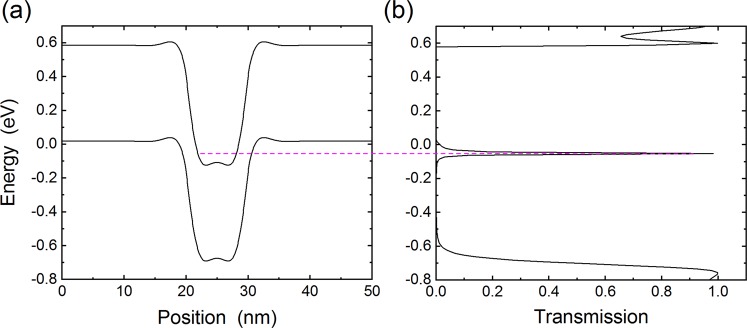
(**a**) Band-bending for the short channel CNT transistor at a gate voltage of +15 V. The Fermi level is at zero. The pink dashed line indicates the lowest energy level. (**b**) The resulting transmission probability shows a sharp peak at the energy of the lowest energy level in the electrostatic quantum dot.

The above calculations suggest that number resolution may be possible by functionalizing a single CNT with several molecules, but the maximum number of resolvable photons may be limited. A potential solution around this problem is to use multiple devices, densely packed within the wavelength of the photon, where each device can respond to only one photon. This leads to the short channel device illustrated in Fig. 2b. We calculated the properties of such a device using our TB-NEGF approach. Figure 8 shows the results of the TB-NEGF approach for a gate voltage of +15 V. There, the large positive gate voltage leads to inversion of the channel from p-type to n-type, creating an electrostatic quantum dot. This leads to quantization of the energy levels inside the dot, with the pink dashed line in Fig. 8a indicating the lowest energy level. We then calculated the electron transmission probability as a function of energy for this band-bending using the NEGF approach. We find a sharp transmission peak at the energy of the lowest quantized level, with a maximum transmission probability of 1, corresponding to the well-known resonant transmission. Therefore, in this regime the conductance is determined by this sharp resonance, and its location with respect to the Fermi level.

**Figure 9:**
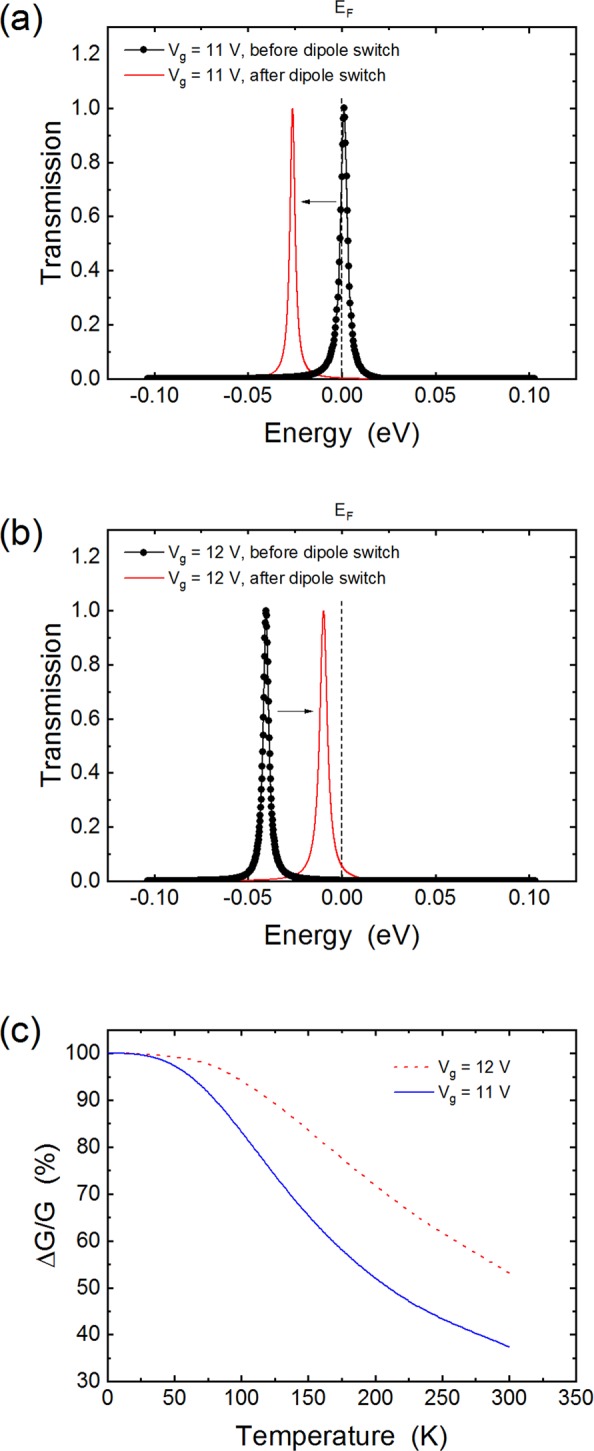
Panels (a) and (b) show the transmission probability for the short channel device at gate voltages of 11 V and 12 V, respectively. In both panels, the Fermi level is at 0. (**c**) Calculated change in conductance as a function of temperature. Here the change in conductance is measured with respect to the largest of the two conductances (i.e. before and after photoswitching).

The sharp transmission opens a route to very sensitive detection since small changes in the electrostatic potential can lead to the transmission resonance moving in and out of the Fermi level. To test this idea, we simulated the system at gate voltages of 11 V and 12 V and changed the dipole moment of a molecule on the surface of the CNT by 1 Debye. Figure 9 shows the impact on the transmission: for both gate voltages, the transmission peak is only 10 meV wide while the shift of the peak is about 25 meV. Therefore, the conductance can vary significantly even at room temperature: as shown in Fig. 9c we obtain changes in conductance of several tens of percent at 300 K. As temperature decreases, the conductance becomes more and more dominated by the transmission at the Fermi level, and the change in conductance reaches 100% at low temperature. This mode of detection would therefore be promising, in particular when dense arrays of individual devices are used to increase the probability of single photon absorption.

### Dark Count Rate

The above calculations establish the typical signal strengths that can be observed from chromophore-functionalized CNTs. To assess whether such signals can be observed it is necessary to also consider the dark count rate (DCR), which is the usual metric for noise in single photon detectors. We obtain the DCR from the electrical transport noise in the CNT, which consists of the thermal (Johnson) noise, the shot noise, and the 1/f noise. Other sources of dark counts would be the thermal excitation of the molecule which would lead to a signal of the same strength as photoexcitation. However, since the energy required to induce the transition is at least 1 eV^30^ and is much larger than the thermal energy even at room temperature, this contribution to dark counts is negligible. Another source of noise is the thermal fluctuations of the molecular dipole orientation; previous calculations^31^ on azobenzenes have shown that the potential energy landscape gives an energy dependence $E(\theta )=0.003{\theta }^{2}$ where *E* is in eV and *θ* is the dipole moment rotation in degrees. From equipartition, this gives average angular fluctuations of 3 degrees at room temperature; for a total dipole moment of 10 Debye this gives variations in electrostatic potential of 5 meV. Since this is a factor of 5 smaller than the change in electrostatic potential from our most conservative calculations (see for example Fig. 7c) we can safely neglect these fluctuations.

The electrical noise follows a Gaussian distribution,

2 $$P(I)=\frac{1}{\sqrt{2\pi {\sigma }^{2}}}{\exp }(-\frac{{I}^{2}}{2{\sigma }^{2}})$$

where

3 $${\sigma }^{2}=4{k}_{B}TG{\tau }^{-1}+2eG{V}_{sd}{\tau }^{-1}+A(G){G}^{2}{V}_{sd}^{2}\,{ln}(\frac{{\tau }_{{\max }}}{\tau }).$$

Here *P(I)* is the probability to observe a current of magnitude *I*, *σ* is the width of the distribution, *G* is the electrical conductance, *τ* is the integration time, τ_max_ is the total duration of the experiment, and V_sd_ is the source-drain bias. The first term in Eq. (3) is the thermal noise, the second term is the shot noise, and the last term is the 1/f noise. Each of these contributions can be obtained by integrating the noise power spectrum up to the frequency f = *τ*^−1^. For the 1/f contribution the current noise power spectrum is given by ${S}_{I}=\frac{A{I}^{2}}{f}=\frac{A{G}^{2}{V}_{sd}^{2}}{f}$ which, when integrated, has a divergence at low frequencies. We regularize the divergence by cutting off the low frequencies with the total duration of the experiment. The pre-factor *A(G)* describes the dependence of the 1/f noise amplitude on the conductance of the CNT; previous theoretical work^32^ obtained excellent agreement with experiment using the expression

4 $$A(G)={\gamma }^{2}{(\frac{dlnG}{d{V}_{g}})}^{2}.$$

A value of γ = 0.1 mV is obtained from the experimental data in ref.^33^, chosen because the CNT transistors in that work achieved near-ideal subthreshold swing of 60 mV/decade, similar to our simulations results in Fig. 4c.

**Figure 10:**
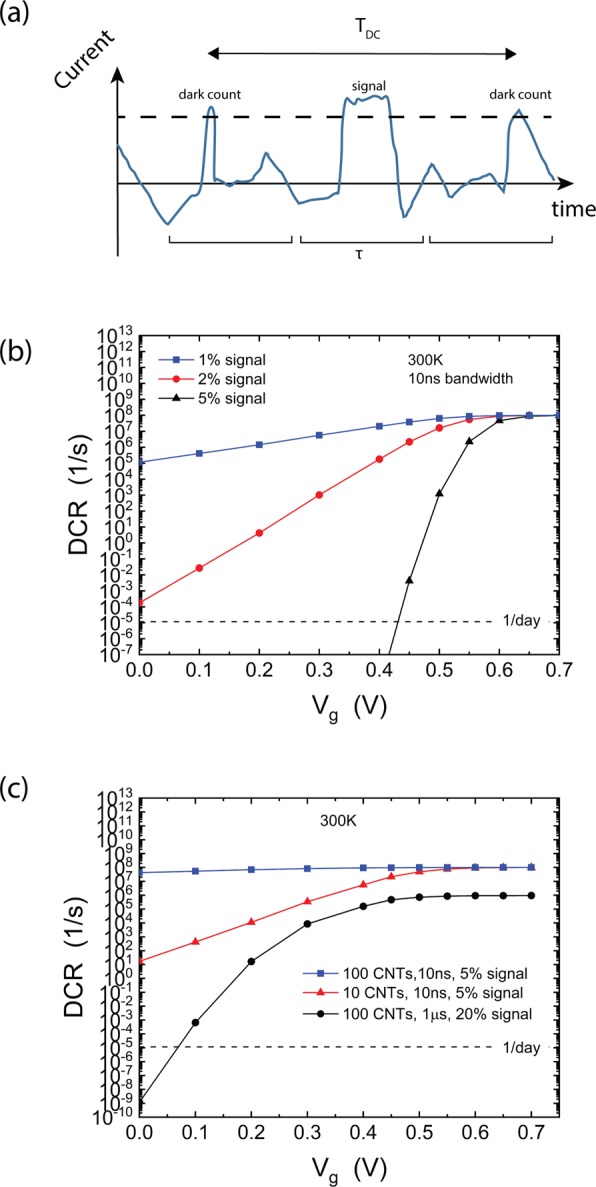
(**a**) Illustration of dark counts originating from electronic noise. The current in the device is integrated over a time window τ, and a hit is recorded when the integrated current exceeds a threshold current I_t_. Dark counts arise when the electronic noise exceeds I_t_; the average separation between such events is denoted T_DC_. (**b**) Calculated dark count rate (DCR) at room temperature for a 10 ns integration window, for the device of Figs 1a and 4c. (**c**) Calculated DCR for a different number of CNTs in parallel. Here the signal strength is 5% or 20% of the conductance of a single CNT.

To obtain the DCR, we use the distribution in Eq. (2) to calculate the average time between dark counts, as illustrated in Fig. 10a. There, we want to detect a signal over a background fluctuating current. To do so, we set a threshold current *I*_*t*_, and register a detection event when the signal is above that threshold. We average the signal over time intervals τ. In some instances, the noise fluctuations will exceed the threshold current, and an event will be recorded even though no photon arrived in that time interval. This corresponds to a dark count. The average time between dark counts *T*_*DC*_ determines the $DCR={T}_{DC}^{-1}$ which is given by

5 $$DCR={\tau }^{-1}{\exp }(-\frac{{I}_{t}^{2}}{2{\sigma }^{2}}).$$

To explicitly calculate the DCR, within the long channel device, we set the threshold current equal to the signal amplitude, which corresponds to the lowest possible DCR. The signal amplitude is taken to be a constant fraction of the device conductance in the dark, at any given gate voltage. This is motivated by the results of Fig. 7d where the fractional change in conductance is relatively constant when compared to the large change of the device conductance. For example, in going from a gate voltage of 0.3 V to a gate voltage of 0.6 V, the signal changes by a factor of two in Fig. 7d but the dark conductance changes by two orders of magnitude (cf. Fig. 4c). In what follows we consider signals that change the conductance by 1%, 2%, and 5%. These values are motivated by the results in Fig. 7. We note that other detection mechanisms (such as charge transfer) may lead to stronger impact on the conductance and could have even better performance.

To illustrate, we first calculated the DCR at room temperature for a source-drain bias of 0.1 V and an integration window of 10 ns. This assumes that the device bandwidth is also equal to 10 ns (the device bandwidth is set by the response time of the molecule and the CNT electronic device and the optimal integration window is equal to the device bandwidth). Figure 10b shows the DCR as a function of gate voltage for different strengths of the signal. For the larger gate voltages, the signal is small due to the low device conductance, so the current threshold *I*_*t*_ has to be low. As a consequence, the detector is swamped by dark counts: the DCR is equal to 1/τ, i.e. a dark count is recorded in every time window. As the gate voltage decreases the device conductance increases, and the signal strength increases as well. The threshold current can be set higher, leading to an exponential decrease of the DCR. The DCR depends sensitively on the strength of the signal: increasing the signal from 1% to 2% already reduces the DCR by orders of magnitude for the lower gate voltages, an effect that is even more prominent for a 5% signal. In fact, for the 5% signal, we find that the DCR can be below 1/day over a range of gate voltages. Note that for a slower detector, the DCR would be lower since the integration time would have to be increased to the detector bandwidth, which reduces the noise according to Eq. (3).

The DCR is also sensitive to the detector architecture. For example, the results of Fig. 10a apply to a device with a single CNT functionalized with one molecule. Since the optical absorption cross-section of such a device is small, it is necessary to either have dense arrays of devices with individual CNTs or to increase the number of CNTs within the same device. To assess the later approach, we calculated the DCR for a device consisting of an array of *N* aligned CNTs, each of the same type. Figure 10c shows that for a 5% signal at 300 K, the device with 10 CNTs already has significantly larger DCR compared to a device with a single CNT. Increasing the number of CNTs to 100 increases the DCR to even larger values. This behavior arises because when a single photon is absorbed, it only changes the conductance of one CNT; therefore, the threshold current in Eq. (4) is unaffected by the number of CNTs in the array. However, according to Eq. (3) the noise fluctuations depend on the total device conductance, which increases with increasing *N*. This increased noise leads to the much larger DCR for arrays. Note that the DCR can still be within reasonable values for slower detectors with stronger signals: indeed, for a 1 µs detector an array of 100 CNTs with 20% signal can still have low DCR. Thus, for this type of architecture, there are tradeoffs between efficiency and dark count rate.

Finally, we note that for the short channel devices, the change in conductance is large (about 40% at room temperature), leading to a low DCR, even for short integration times and a large number of CNTs. For example, a device with 200 CNTs and 1 ns bandwidth would have a DCR ~ 1/day.

## Conclusion

In conclusion, we find that devices utilizing chromophore-functionalized carbon nanotubes are promising for single photon detection. Even under modest assumptions for changes in the molecular dipole moment, changes in conductance of a few percent to tens of percent are possible depending on the device channel length. Furthermore, these devices may provide new approaches for photon number resolution, for example by having multiple molecules on the same CNT. The device sensitivity is accompanied by regimes of operation where the dark count rate satisfies some of the stringent requirements of photodetector technology. We anticipate that experimental realization of these devices will highlight additional challenges such as the response speed, additional fluctuations in the molecule position and its dipole moment. In addition, other sensing mechanisms such as charge transfer between the molecule and the CNT may lead to even larger changes in conductance and better performance. Taken as a whole, our work establishes a path toward realizing bio-inspired single photodetectors with attractive performance.
